# A novel *COL4A1* frameshift mutation in familial kidney disease: the importance of the C-terminal NC1 domain of type IV collagen

**DOI:** 10.1093/ndt/gfw051

**Published:** 2016-04-08

**Authors:** Daniel P. Gale, D. Deren Oygar, Fujun Lin, P. Derin Oygar, Nadia Khan, Thomas M.F. Connor, Marta Lapsley, Patrick H. Maxwell, Guy H. Neild

**Affiliations:** 1 UCL Centre for Nephrology, University College, London, UK; 2 Nephrology Department, Nicosia State Hospital, Nicosia, North Cyprus; 3 Department of Nephrology, Xin Hua Hospital, School of Medicine, Shanghai Jiao Tong University, Shanghai, China; 4 Department of Pediatrics, Nicosia State Hospital, Nicosia, North Cyprus; 5 West London Renal and Transplant Institute, Imperial College, London, UK; 6 South West Thames Institute for Renal Research, St Helier Hospital, Carshalton, UK; 7 School ofClinical Medicine, Cambridge University, Cambridge, UK

**Keywords:** *COL4A1*, familial nephropathy, genetic renal disease, glomerular basement membrane, type IV collagen

## Abstract

**Background:**

Hereditary microscopic haematuria often segregates with mutations of *COL4A3*, *COL4A4* or *COL4A5* but in half of families a gene is not identified. We investigated a Cypriot family with autosomal dominant microscopic haematuria with renal failure and kidney cysts.

**Methods:**

We used genome-wide linkage analysis, whole exome sequencing and cosegregation analyses.

**Results:**

We identified a novel frameshift mutation, c.4611_4612insG:p.T1537fs, in exon 49 of *COL4A1*. This mutation predicts truncation of the protein with disruption of the C-terminal part of the NC1 domain. We confirmed its presence in 20 family members, 17 with confirmed haematuria, 5 of whom also had stage 4 or 5 chronic kidney disease. Eleven family members exhibited kidney cysts (55% of those with the mutation), but muscle cramps or cerebral aneurysms were not observed and serum creatine kinase was normal in all individuals tested.

**Conclusions:**

Missense mutations of *COL4A1* that encode the CB3 [IV] segment of the triple helical domain (exons 24 and 25) are associated with HANAC syndrome (hereditary angiopathy, nephropathy, aneurysms and cramps). Missense mutations of *COL4A1* that disrupt the NC1 domain are associated with antenatal cerebral haemorrhage and porencephaly, but not kidney disease. Our findings extend the spectrum of *COL4A1* mutations linked with renal disease and demonstrate that the highly conserved C-terminal part of the NC1 domain of the α1 chain of type IV collagen is important in the integrity of glomerular basement membrane in humans.

## INTRODUCTION

Isolated microscopic haematuria is a significant risk factor for kidney failure in later life [1] and can be the presenting feature of a number of disorders, which can be monogenic or non-familial. Since kidney biopsy is seldom performed where there is no evidence of kidney damage (i.e. proteinuria, hypertension or renal impairment), a firm diagnosis has traditionally only been possible in patients with more advanced kidney disease. Today, however, the cause of familial kidney disease may be identified at an early stage using genetic testing. This can have important implications for the patient and his/her family because prognosis and risk to family and offspring depends on the diagnosis [2].

**FIGURE 1:**
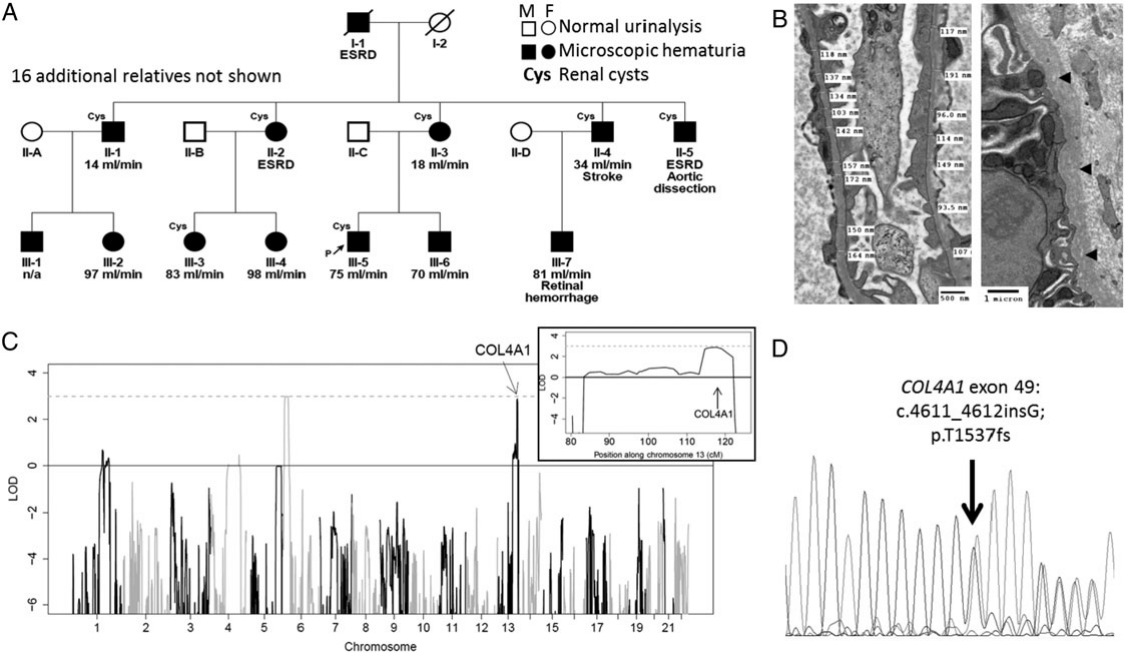
(**A**) Family tree showing affected individuals participating in the genome-wide linkage study. P denotes proband. Estimated glomerular filtration rate in mL/min is shown for each individual (where known) and ESRD denotes end-stage renal disease. (**B**) Electron micrographs of kidney biopsy from individual III-5 showing thin glomerular basement membranes and lamellation and lucencies in the tubular basement membranes (arrowheads). (**C**) Genome-wide linkage excluded loci containing genes previously associated with haematuria, except for a locus on chromosome 13 that contains the *COL4A1* gene (inset), LOD score = 3. (**D**) Sanger sequencing of exon 49 of *COL4A1* demonstrated a G insertion, predicting a p.T1537fs mutation of the type IV collagen α1 chain.

The most common inherited cause of microscopic haematuria is thin basement membrane nephropathy (TBMN), which is characterized by thinning or irregularity of the glomerular basement membrane visible on electron microscopy. In many families a cause is not identified, however, in 40–50% cases, TBMN is linked with a heterozygous mutation of the *COL4A3, COL4A4* or *COL4A5* genes that encode type IV collagen, and the clinical features overlap with the carrier state for autosomal recessive or X-linked Alport syndrome [3–5]. Most carriers of a heterozygous *COL4A3/4* mutation have an excellent prognosis; however, 15–20% will develop renal impairment or even end-stage renal disease (ESRD), usually in later life [4, 6]. Other monogenic causes of kidney disease that can present with isolated microscopic haematuria include Alport syndrome, which is either X-linked (caused by hemizygous mutation of *COL4A5*) or autosomal recessive (caused by bi-allelic mutation of the *COL4A3* or *COL4A4* genes); CFHR5 nephropathy (caused by an internal duplication of *CFHR5* [7]); MYH9-associated glomerulopathy (caused by mutations of *MYH9* and also associated with proteinuria, deafness and platelet abnormalities [8]); and hereditary angiopathy, nephropathy, aneurysms and cramps (HANAC) syndrome, which is associated with heterozygous mutations in exons 24 and 25 of the *COL4A1* gene [9]. We investigated a family of Turkish Cypriot origin in which microscopic haematuria and renal impairment segregated as a dominant trait (Figure 1).

## MATERIALS AND METHODS

All research involving human participants was performed with written informed consent and was approved by the ethics committee of Lefkosa Burhan Nalbantoğlu State Hospital. All participants provided informed consent for their involvement in the research in accordance with the Declaration of Helsinki and for publication of the study results. Individuals were genotyped on the Linkage IV panel (Illumina, San Diego, CA, USA) according to the manufacturers' instructions. Linkage analysis was performed using GeneHunter v2.1 [10]. Exome sequencing was performed by enrichment with the Agilent SureSelect All Exon array and sequencing using an Illumina Genome Analyzer IIx. Reads were aligned to the reference genome (GRCh37) using Novoalign (Novocraft Technologies, Selangor, Malaysia). Variants were called using GATK [11] and annotated with Annovar [12]. Resequencing of *COL4A1* exon 49 was performed using the primers 5′-TGCTTGTTGTCCAGCTGAAAT-3′ and 5′-ACAGGAGGAAAGGCAACCAC-3′, amplified by polymerase chain reaction at an annealing temperature of 58°C. Three-dimensional models were generated using Jmol [13], based on the crystal structure 1li1 from the protein database [14].

Retinol-binding protein (RBP) was assayed as previously described using a polyclonal in-house sandwich ELISA [15]. Urinary N-acetyl-beta-D-glucosaminidase (NAG) activity was measured using a kit from PPR Diagnostics (London, UK). Albumin and creatinine (enzymatic) were measured on an Abbott Architect (Abbott Diagnostics, Lake Forest, IL, USA).

## RESULTS

Clinical information was available for 29 family members, of whom 19 exhibited >1+ microscopic haematuria on urine dipstick testing. Fifteen family members had renal impairment [(estimated glomerular filtration rate eGFR) <90 mL/min], three requiring renal replacement therapy.

### Genotype

Genes associated with autosomal dominant thin basement membrane nephropathy (*COL4A3* and *COL4A4*) were sequenced in the proband by amplification of each exon and Sanger sequencing. This did not reveal any rare or likely pathogenic variants. A genome-wide linkage study was performed using DNA from 12 affected family members. This excluded linkage with loci containing the genes *COL4A3, COL4A4, COL4A5, MYH9* and *CFHR5* (LOD < −2 at each locus), but did demonstrate linkage (LOD = 3) with loci on chromosomes 6 and 13 that include the *COL4A1* gene on chromosome 13 (Figure 1C). Whole exome sequencing was performed in the proband, which identified no likely pathogenic variants in *COL4A3*, *COL4A4*, *COL4A5*, *MYH9* or *CFHR5*. Across the whole exome, 688 variants that were both rare (i.e. occur with an allele frequency of <0.5% in the 1000 genomes database) and predicted a change in amino acid sequence of a protein (including amino acid substitutions, insertions, deletions, splicing, frameshift or termination mutations) were identified. Of these, 644 were heterozygous and 270 were novel (i.e. not reported in dbSNP). Two rare variants, both heterozygous, occurred within the linked loci. One variant predicted a missense mutation p.A101T in *TMEM14C* (NM_016462) on chromosome 6. This gene has not previously been associated with kidney disease and the variant is predicted to be benign, with SIFT and Polyphen scores of 0.42 and 0.023, respectively. The other variant was in the *COL4A1* gene and encodes a novel frameshift mutation c.4611_4612insG; p.T1537fs (transcript NM_00185). This was considered a good candidate for causing disease since other mutations in this gene are associated with HANAC syndrome, in which haematuria, kidney cysts and renal impairment are features.

The *COL4A1* mutation was confirmed by Sanger sequencing (Figure 1D), and screening across all available samples revealed its presence in all affected individuals participating in the linkage study and in a further eight relatives, seven of whom had one or more of haematuria, cysts or evidence of reduced renal function (eGFR <90 mL/min). The individual lacking clear evidence of kidney disease was 23 years old at the time of assessment and had trace haematuria on dipstick testing. Eight offspring of affected family members were found not to harbour the *COL4A1* variant, of whom one was found to have dipstick haematuria on a single occasion, associated with normal renal function and imaging, and one (designated individual II-6) exhibited renal impairment, haematuria, proteinuria and kidney cysts (Table 1). We regard these individuals as phenocopies, which is not an unexpected finding given the high prevalence of clinical evidence of kidney disease known to be present in this and other populations [16–18]. The mutation is not present in any online databases of human genetic variation and was not detected by sequencing a cohort of 63 unrelated healthy Turkish Cypriot adults.

**Table 1.** Clinical features in family members with and without the *COL4A1* p.T1537fs frameshift mutation

Clinical feature		With mutation	Without mutation
Haematuria	Present	17	2
	Absent/trace	2	6
Proteinuria	Present	7	1
	Absent	12	7
Kidney cysts	Present	11	1
	Absent	7	4
Creatine kinase	Elevated	0	0
	Normal	16	6
Retinal vessels	Tortuous	0	0
	Normal	4	0
Kidney function	eGFR > 90	5	7
	eGFR 31–90	14	1
	eGFR < 30	4	0
	ESRD	3	0
Total family members		20	8

Proteinuria: albumin:creatinine ratio >3.0 mg/mmol; kidney cysts: as seen on renal ultrasound; retinal vessels: as seen by retinal photography.

To determine whether an additional mutation might be segregating in other family members we went on to sequence *COL4A3*, *COL4A4* and *COL4A5* in individual II-2 (who harboured the *COL4A1* mutation and had developed ESKD) and individual II-6. No rare or likely pathogenic mutations of these genes were identified in either of these individuals. We conclude that the *COL4A1* mutation is responsible for the familial renal disease in this kindred. We went on to examine whole exome sequencing data from four other families in which glomerular basement membrane abnormalities and microscopic haematuria segregated as a dominant trait in the absence of a *COL4A3*, *COL4A4* or *COL4A5* mutation but did not find any additional likely pathogenic variants in *COL4A1*.

### Clinical phenotype

Renal failure was unpredictable, with creatinine rising above the normal range in some affected members after the age of 40 years. End-stage renal failure ranged from 63 to 70 years in three of nine siblings in the first generation. In those affected, one or more kidney cysts were detectable by ultrasound from the age of 40 years in 12 family members. Hypertension was variable, but could be present from 35 years of age. No family members reported a history of muscle cramps or cerebral aneurysms, and serum creatine kinase was normal in all 22 family members tested. Some family members had evidence of vascular disease (summarized in Figure 1A). Hyperuricaemia was not found and no patient had a history of gout. Brain MRI scanning and retinal photography in four affected members of the family revealed no evidence of cerebral or ocular angiopathy.

Little or no proteinuria (albumin:creatinine ratio <3 mg/mmol) was found until the eGFR was <50 mL/min/1.73 m^2^. Urine samples from 15 affected people were also investigated for tubular proteinuria. Of the 10 mutation carriers without microalbuminuria, 5 had an elevated RBP:creatinine ratio ≥25 mg/mmol (97.5th upper centile <18 mg/mmol creatinine) and 3 had an elevated NAG:creatinine ratio (normal range <28 µmol/h/L).

A kidney biopsy was performed in one family member at age 47 years to investigate familial nephropathy and reduced eGFR. Light microscopic examination was normal with the exception of minimal global glomerulosclerosis (1 of 25 glomeruli) and <1% tubular atrophy. Immunostains for type IV collagen were not performed, but stains for immunoglobulins and complement were negative. Electron microscopy demonstrated areas of thinning of glomerular basement membranes (down to 93.5 nm) and subtle lamellation of tubular basement membranes (Figure 1B). Glomerular basement membrane splitting or irregularity of the outer surface was not observed and the ultrastructural appearances were judged to represent thin basement membrane nephropathy rather than Alport syndrome.

## DISCUSSION

Type IV collagen is an important constituent of the extracellular matrix and forms a complex meshwork that provides structural integrity to basement membranes [19]. Individual type IV collagen molecules are made up of three alpha chains that form a heterotrimeric structure that includes a non-collagenous (NC1) domain at the C-terminus that initiates heterotrimer formation [20], and a long collagenous ‘tail’ interspersed with non-collagenous interruptions. The collagenous domains of three alpha chain subunits interweave to form a stiff triple helical structure and the non-collagenous interruptions likely confer flexibility and allow intermolecular cross-linking, leading to formation of the macromolecular collagen network [21]. The NC1 domains of adjacent trimers are themselves covalently cross-linked by sulfilimine bonds at conserved residues to form hexamers [22]. The α1.α1.α2 isoform of type IV collagen is composed of two α1 chains and one α2 chain, encoded by the genes *COL4A1* and *COL4A2*, respectively, and is very highly conserved across species, forming an important component of most basement membranes. This contrasts with the other naturally occurring type IV collagen isoforms (α3.α4.α5 and α5.α5.α6) that have a more restricted distribution of expression and are less broadly conserved across species [21].

Pathogenic mutations of *COL4A1* are associated with neurological, vascular and renal disorders. Previously described phenotypes cluster into primarily neurological disease, in which porencephaly (the occurrence of fluid filled cavities in the brain) and cerebral vasculopathy or haemorrhage cause significant neurological damage early in life [23], and HANAC syndrome, in which neurological involvement tends to be milder and haematuria, kidney cysts and occasionally late-onset renal impairment are features [9]. The mechanisms of pathogenicity in *COL4A1*-associated diseases are incompletely understood. However, it is unlikely that haplo-insufficiency is a major contributor in most cases: previously described pathogenic mutations in humans are almost all missense variants and do not include early nonsense mutations or heterozygous whole gene deletions; only a single collagenous-domain frameshift mutation that reduced transcript levels has been reported to date [23]. Moreover, mice heterozygous for a null *Col4A1* allele do not display any detectable phenotype [21]. Mutations causing HANAC syndrome have been reported only in exons 24 and 25 of *COL4A1*, encoding a region of the protein that contains multiple potential integrin-binding sites, and it has been suggested that disruption of normal interaction between type IV collagen and integrins might be responsible for HANAC syndrome [21, 24]. Thinning of glomerular basement membranes (in which α3.α4.α5 is the predominant type IV collagen isoform) is not reported in either HANAC syndrome or other *COL4A1*-associated diseases.

**FIGURE 2:**
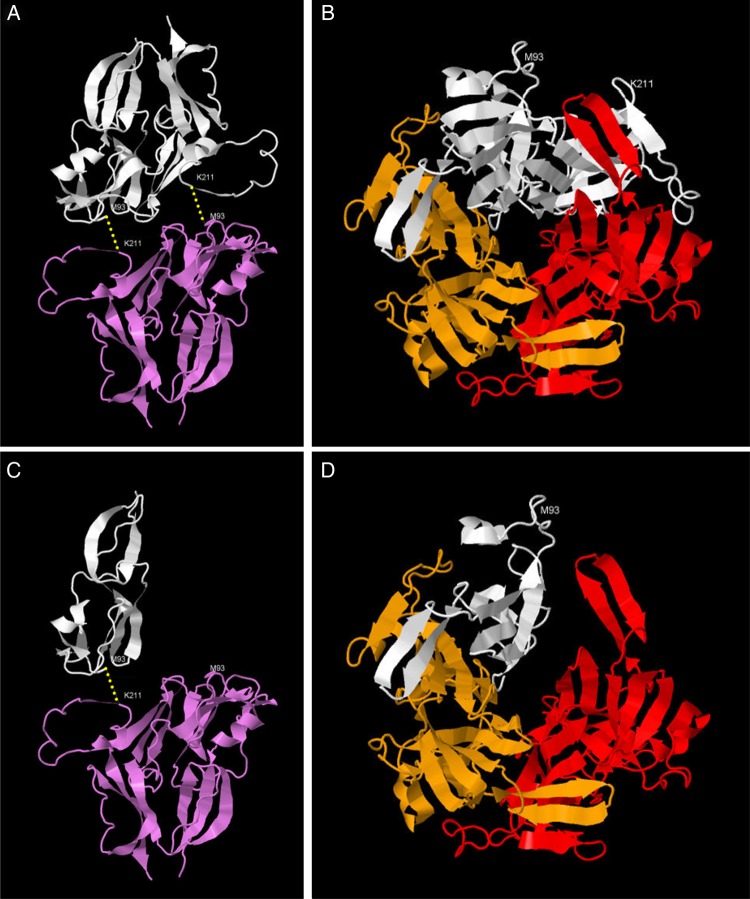
(**A**) The NC1 domain of one α1 chain (white) of a trimeric type IV collagen molecule forms two sulfilimine bonds (yellow) with the NC1 domain of an α1 chain of the adjacent trimer (violet). Additional alpha chains in each trimer not shown. (**B**) The NC1 domains of two α1(IV) chains (white and orange) and one α2(IV) chain (red) interact to form a heterotrimer, viewed face on to the dimerization surface [i.e. perpendicular to the view shown in (A)]. (**C**) The truncated protein predicted by the p.T1537fs mutation lacks the C-terminal K^211^ residue and hence is only able to form a single sulfilimine bond. (**D**) Truncation of the protein is predicted to disrupt the normal interaction with one other subunit of a type IV collagen heterotrimer, although the surface interacting with the other subunit is intact.

Frameshift variants of *COL4A1* are exceedingly rare, with only one example observed (encoding p.P438fs) in >120 000 alleles tested in the ExAC project and a single pathogenic allele (p.G696fs) reported previously in the medical literature [23]. The p.T1537fs mutation we identified is predicted to result in the substitution of the 132 C-terminal amino acids of the protein with the peptide ‘GGGKHKTIY’ followed by a premature termination codon. The missing C-terminal domain is highly conserved across evolution (present even in cnidarian and nematode species; see Table 2) and includes Lysine 211 (K^211^), which is essential for the sulfilimine bond cross-linking adjacent trimers to form hexameric type IV collagen [25]. It also encodes the part of the protein that interacts with one (but not both) of the other subunits in a heterotrimer (Figure 2).

**Table 2.** The C-terminal NC1 domain of *COL4A1* is highly conserved across species

Common name	Phylum	Latin name	Sequence
Mutant allele			TPEPMPMS**M**APITGGGKHKTIY
Human	Chordata	*Homo sapiens*	TPEPMPMS**M**APITGENIRPFISRCAV…PTPSTL**K**AGELRTHVSRCQVCMRRT
Chimpanzee	Chordata	*Pan troglodytes*	TPEPMPMS**M**APITGENIRPFISRCAV…PTPSTL**K**AGELRTHVSRCQVCMRRT
Monkey	Chordata	*Macaca mulatta*	TPEPMPMS**M**APITGDNIRPFISRCAV…PTPSTL**K**AGELRTHVSRCQVCMRRT
Wolf	Chordata	*Canis lupus*	TPEPMPMS**M**APIAGDNIRPFISRCAV…PTPSTL**K**AGELRTHVSRCQVCMRRT
Cow	Chordata	*Bos taurus*	TPEPMPMS**M**APITGENIRPFISRCAV…PTPSTL**K**AGELRTHVSRCQVCMRRT
Mouse	Chordata	*Mus musculus*	TPEPMPMS**M**APISGDNIRPFISRCAV…PTPSTL**K**AGELRTHVSRCQVCMRRT
Rat	Chordata	*Rattus norvegicus*	TPEPMPMS**M**APISGDNIRPFISRCAV…PTPSTL**K**AGELRTHVSRCQVCMRRT
Chicken	Chordata	*Gallus gallus*	TPEPMPMS**M**APITGESIRPFISRCSV…PTPSTL**K**AGDLRSNVSRCQVCMRNT
Zebrafish	Chordata	*Danio rerio*	TPEPMPMS**M**APVTGESIKPFISRCAV…PVPATL**K**AGSLRTHISRCQVCMKRV
Xenopus	Chordata	*Xenopus tropicalis*	TPEPMPMS**M**APITGDGIKPFISRCTV…PTPSTL**K**AGDLRTHVSRCQVCMRRTS
Fruit Fly	Arthropoda	*Drosophila melanogaster*	T-TNAAIP**M**MPVENIEIRQYISRCVV…PQQQTI**K**AGERQSHVSRCQVCMKNSS
Roundworm	Nematoda	*Ascaris suum*	LSTTAPIP**M**MPVSEGGIEPYISRCAV…PESETL**K**AGSLRTRVSRCQVCIRSPDVQPYRG
Roundworm	Nematoda	*Caenorhabditis elegans*	TDEPMTPM**M**NPVTGTAIRPYISRCAV…PMSQTL**K**AGGLKDRVSRCQVCLKNR
Pink Hydroid	Cnidaria	*Ectopleura larynx*	TPEPMPMM**M**NPVEGRDIEKYVSRCSV…PQSQTL**K**AGNQRSRISRCQVCMRRR
Freshwater Jellyfish	Cnidaria	*Craspedacusta sowerbyi*	STEQEPNM**M**RPSKGIENRDYISRCIV…PVPETI**K**AGQLRQRVSRCAVCMKNNKANEP

The residues (M^93^ and K^211^) that participate in the sulfilimine bond between adjacent heterotrimers are in bold. Approximately 100 residues (denoted by ‘…’) are omitted for clarity. The mutant terminal peptide present in the family is underlined and lacks K^211^. Adapted from Fidler *et al.* [25].

One possible mechanism whereby loss of this part of the NC1 domain could cause disease includes disruption of sulfilimine bond(s) by inclusion of one or more defective α1 chain missing the C-terminal part of its NC1 domain within an α1.α1.α2 type IV collagen heterotrimer (Supplementary Video S1). Alternatively, since it is known that α1 type IV chains are expressed in podocytes and present in glomeruli of healthy adults [26, 27], it is possible that the defective NC1 domain resulting from this mutation could cause incorrect recognition and assembly of glomerular type IV collagen heterotrimers [20], perhaps resulting in incorporation of the mutant α1 subunit in α3.α4.α5 type IV collagen trimers of glomerular basement membranes. This might explain the observed thinning of glomerular basement membranes. Our data clearly do not exclude a dominant negative or gain-of-function effect of the mutation mediated by a mechanism unrelated to trimer formation, such as interaction of monomeric mutant protein with type IV collagen via the non-collagenous interruptions within the alpha chains. The possibility that the disease mechanism is disruption of basement membrane formation due to haplo-insufficiency for *COL4A1* is considered less likely for the reasons stated above. A homozygous truncating mutation within the C-terminal NC1 domain of *COL4A4* has previously been reported in association with autosomal recessive Alport syndrome, but the phenotype of the obligate carrier parents was not documented in this report [28], so it is not known whether this similar mutation in a different type IV collagen chain in heterozygosity has similar phenotypic effects to the mutation described here.

In summary, we present a novel *COL4A1* mutation linked with kidney disease that is predicted to cause loss of a highly conserved part of the C-terminal NC1 domain of the α1 type IV collagen chain that is important in interactions within and between type IV collagen heterotrimers. This demonstrates that this part of the protein is important in the normal function of type IV collagen in humans.

## SUPPLEMENTARY DATA

Supplementary data are available online at http://ndt.oxfordjournals.org.

## CONFLICT OF INTEREST STATEMENT

The authors have no competing interests. The results presented in this paper have not been published previously in whole or part, except in abstract format.

(See related article by Savige. A further genetic cause of thin basement membrane nephropathy. *Nephrol Dial Transplant* 2016; 31: 1758–1760)

## Supplementary Material

Supplementary Data
