# Supplementary material for: A novel COL4A1 frameshift mutation in familial kidney disease: the importance of the C-terminal NC1 domain of type IV collagen
Source: Nephrol Dial Transplant. 2016 Apr 8;31(11):1908–14. doi: 10.1093/ndt/gfw051 (PMC5091614; doi:10.1093/ndt/gfw051)
Supplement: Supplementary Data [file supp_31_11_1908__index.html]

A novel COL4A1 frameshift mutation in familial kidney disease: the importance of the C-terminal NC1 domain of type IV collagen — A novel COL4A1 frameshift mutation in familial kidney disease: the importance of the C-terminal NC1 domain of type IV collagen — Supplementary Data 

# A novel *COL4A1* frameshift mutation in familial kidney disease: the importance of the C-terminal NC1 domain of type IV collagen

## Supplementary Data

Supplementary Data

- Supplementary Data - mp4 file
